# The 1-Tosylpentan-3-one Protects against 6-Hydroxydopamine-Induced Neurotoxicity

**DOI:** 10.3390/ijms18051096

**Published:** 2017-05-19

**Authors:** Chien-Jen Kao, Wu-Fu Chen, Bo-Lin Guo, Chien-Wei Feng, Han-Chun Hung, Wen-Ya Yang, Chun-Sung Sung, Kuan-Hao Tsui, Hsin Chu, Nan-Fu Chen, Zhi-Hong Wen

**Affiliations:** 1 Department of Marine Biotechnology and Resources, National Sun Yat-sen University, Kaohsiung 804, Taiwan; kaochienjen@yahoo.com.tw (C.-J.K.); ma4949@cgmh.org.tw (W.-F.C.); askedc321@gmail.com (B.-L.G.); qscjuejuejue@gmail.com (C.-W.F.); hanchun25@gmail.com (H.-C.H.); s8222889@gmail.com (W.-Y.Y.); 2 Department of Internal Medicine, Gangshan Branch of Armed Forces Kaohsiung General Hospital, No. 1, Dayi 2nd Rd., Gangshan Dist., Kaohsiung City 820, Taiwan; 3 National Defense Medical Center, Department of Internal Medicine of Gangshan Branch of Kaohsiung Armed Forces General Hospital, Kaohsiung 82049, Taiwan; hrchu@mail.ndmctsgh.edu.tw; 4 Department of Neurosurgery, Kaohsiung Chang Gung Memorial Hospital and Chang Gung University College of Medicine, Kaohsiung 804, Taiwan; 5 Doctoral Degree Program in Marine Biotechnology, National Sun Yat-sen University, 70 Lien-Hai Road, Kaohsiung 804, Taiwan; 6 Doctoral Degree Program in Marine Biotechnology, Academia Sinica, 128 Academia Road, Section 2, Nankang, Taipei 115, Taiwan; 7 Department of Anesthesiology, Taipei Veterans General Hospital, No. 201, Section 2, Shipai Road, Taipei 11217, Taiwan; sung6119@gmail.com; 8 School of Medicine, National Yang-Ming University, No. 155, Section 2, Linong Street, Taipei 11221, Taiwan; 9 Department of Obstetrics and Gynecology, Kaohsiung Veterans General Hospital, Kaohsiung 804, Taiwan; khtsui60@gmail.com; 10 Department of Obstetrics and Gynecology and Institute of Clinical Medicine, National Yang-Ming University, Taipei 112, Taiwan; 11 Department of Biological Science, National Sun Yat-sen University, Kaohsiung 804, Taiwan; 12 Department of Pharmacy and Master Program, College of Pharmacy and Health Care, Tajen University, Pingtung 900, Taiwan; 13 Division of Neurosurgery, Department of Surgery, Kaohsiung Armed Forces General Hospital, Kaohsiung 804, Taiwan; 14 Department of Neurological Surgery, Tri-Service General Hospital, National Defense Medical Center, Taipei 114, Taiwan

**Keywords:** neuroprotection, 6-OHDA-induced apoptosis, marine compounds, zebrafish, SH-SY5Y cells, 1-tosylpentan-3-one

## Abstract

Previous studies have demonstrated that the marine compound austrasulfone, isolated from the soft coral *Cladiella australis*, exerts a neuroprotective effect. The intermediate product in the synthesis of austrasulfone, dihydroaustrasulfone alcohol, attenuates several inflammatory responses. The present study uses in vitro and in vivo methods to investigate the neuroprotective effect of dihydroaustrasulfone alcohol-modified 1-tosylpentan-3-one (1T3O). Results from in vitro experiments show that 1T3O effectively inhibits 6-hydroxydopamine-induced (6-OHDA-induced) activation of both p38 mitogen-activated protein kinase (MAPK) and caspase-3 in SH-SY5Y cells; and enhances nuclear factor erythroid 2–related factor 2 (Nrf2) and heme oxygenase-1 (HO-1) expression via phosphoinositide 3-kinase (PI3K)/protein kinase B (Akt) signaling. Hoechst staining and Terminal deoxynucleotidyl transferase dUTP nick end labeling (TUNEL) staining results reveal that 1T3O significantly inhibits 6-OHDA-induced apoptosis. In addition, the addition of an Akt or HO-1 inhibitor decreases the protective effect of 1T3O. Thus, we hypothesize that the anti-apoptotic activity of 1T3O in neuronal cells is mediated through the regulation of the Akt and HO-1 signaling pathways. In vivo experiments show that 1T3O can reverse 6-OHDA-induced reduction in locomotor behavior ability in zebrafish larvae, and inhibit 6-OHDA-induced tumor necrosis factor-alpha (TNF-α) increase at the same time. According to our in vitro and in vivo results, we consider that 1T3O exerts its anti-apoptotic activities at SH-SY5Y cells after 6-OHDA challenges, probably via the regulation of anti-oxidative signaling pathways. Therefore, this compound may be a promising therapeutic agent for neurodegenerations.

## 1. Introduction

Parkinson’s disease (PD) is a common neurodegenerative disease (ND) whose global incidence is second only to that of Alzheimer’s disease (AD) [1,2]. The primary pathological characteristic of PD is the extensive death of dopaminergic neurons in the substantia nigra pars compacta [3], resulting in physical disability and resting tremors. Studies have suggested that pathological mechanisms such as oxidative stress, mitochondrial dysfunction, inflammatory process, and ubiquitin-proteasome system dysfunction, may be associated with the death of dopaminergic neurons in the substantia nigra pars compacta of PD-affected brains [4,5,6].

Neuroinflammation is believed to be associated with PD [7,8,9,10]. Multiple inflammatory cytokines, such as tumor necrosis factor-alpha (TNF-α), participate in the inflammatory response, and long-term inflammation results in oxidative stress and destruction of brain matter [11,12,13]. A previous clinical study found that oxidative stress is a major cause of gradual death of substantial nigra cells in the brain [5]. Dopamine metabolism involves an oxidoreductive reaction where the metabolic byproduct, hydrogen peroxide, induces oxidative stress in cells [14,15]. The antioxidant response element (ARE) pathway is one of the key mechanisms for countering oxidative stress [16]. The most widely known ARE-associated transcription factor is nuclear factor erythroid 2–related factor 2 (Nrf2). Upon activation, Nrf2 separates from the kelch-like ECH-associated protein 1 (Keap1) [17] and enters the nucleus to bind with AREs to form a transcription complex [18], resulting in the production of a variety of antioxidant-related factors, including heme oxygenase-1 (HO-1) and superoxide dismutase (SOD) [19]. A previous study using *Drosophila* models of PD confirmed that activated Nrf2 signaling can effectively inhibit α-synuclein-induced locomotion defects and restore the expression level of tyrosine hydroxylase (TH), a marker of dopaminergic neurons [19]. Furthermore, a number of PD-related studies have emphasized the importance of Nrf2 pathway regulation in neuroprotective activity [20,21,22,23].

Several groups have attempted to regulate cell-survival signaling pathways to achieve neuroprotection. The phosphoinositide 3-kinase (PI3K)/protein kinase B (Akt) signaling pathway plays a critical role in ensuring cell growth and viability [24]. Akt phosphorylation can promote the activation of the downstream Nrf2/HO-1 antioxidant pathway to protect cells from oxidative stress, and promote the activation of B-cell lymphoma-extra-large (Bcl-xL) to inhibit the apoptosis factor, caspase-3 [25]. A study showed that the clinical drug selegiline can effectively reverse neurotoxin-induced decrease in Akt phosphorylation and activate the downstream Nrf2/HO-1 pathway [26], thereby protecting damaged dopaminergic neruons [27,28,29]. Extracellular signal-regulated kinase (ERK) is a member of the mitogen-activated protein kinase (MAPK) family, and the ERK signaling pathway plays a key role in mitosis regulation, cell growth, and apoptosis inhibition [30]. Phosphorylated ERK promotes the entry of transcription factors Nrf2 and cyclic adenosine monophosphate (cAMP) responsive element binding protein (CREB) into the nucleus to bind to ARE, cAMP responsive element (CRE), and other gene sequences to promote the expression of antiapoptotic and antioxidant proteins [31]. A previous study showed that activated ERK can inhibit 6-hydroxydopamine (6-OHDA)-induced apoptosis of neuronal cells and reverse decreases in B-cell lymphoma 2 (Bcl-2) protein expression [32]. However, the function of activated p38 MAPK, another member of the MAPK family, differs significantly from that of ERK. Studies showed that bacterial lipopolysaccharide (LPS) causes an increase in oxidative stress in microglia as well as an increase in the expression of p38 MAPK, resulting in cell death [30,33]. Phosphorylated p38 promotes the release of cytochrome c into the cytoplasm and activates caspase 3, causing mitochondrial damage and neuronal death [34].

**Figure 1:**
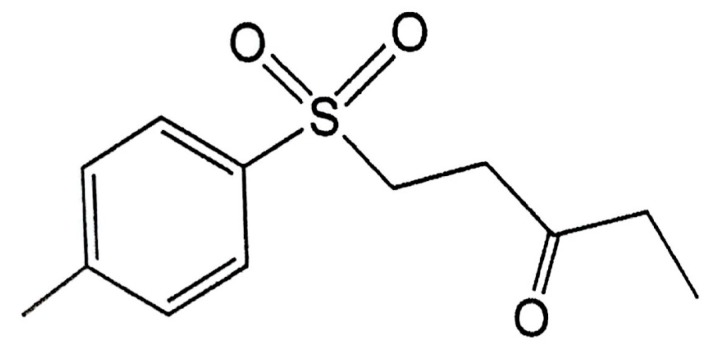
Chemical structure of 1-tosylpentan-3-one (1T3O).

A previous study found that the marine compound austrasulfone, could be extracted from *Cladiella australis*, a species of soft coral collected from Taiwan waters. Austrasulfone effectively decreases 6-OHDA-induced SH-SY5Y cell death. Furthermore, a previous study found that the intermediate product in the synthesis of austrasulfone, dihydroaustrasulfone alcohol, decreases the expression of proinflammatory cytokines such as inducible nitric oxide synthase (iNOS) and cyclooxygenase-2 (COX2), thus alleviating neuropathic pain and multiple sclerosis in *rats* regulating lipid metabolism in macrophages, and inhibiting restenosis [35,36,37,38]. The above results suggest that the bioactivity of austrasulfone and its intermediate, dihydroaustrasulfone, could be possibly related to their structures. To increase the penetration of these compounds through the cell membrane, our group modified the polar hydroxyl group of the dihydroaustrasulfone alcohol into a benzene ring to produce 4-(phenylsulfanyl) butane-2-one (4-PSB-2). A study showed that 4-PSB-2 has a neuroprotective effect in a rat model of optic nerve crush. A subcutaneous injection of 4-PSB-2 immediately after injury can attenuate the death of retinal ganglion cells induced by crush injury [39]. In addition, it was found that 4-PSB-2 could inhibit melanin synthesis and melanosome maturation to produce a whitening effect [40]. Herein, on the basis of an educated guess, we combined the structures of the three compounds described above and synthesized 1-tosylpentan-3-one (1T3O; Figure 1). This study adopted in vitro and in vivo 6-OHDA-induced PD models to evaluate the expression of apoptotic, oxidative stress, and inflammatory markers, and investigate the neuroprotective effect of the dihydroaustrasulfone alcohol derivative, 1T3O (Figure 1). On the basis of neuroprotective efficacy results in this study, we hope to promote the application of marine compounds in the treatment of NDs and development of potential drugs.

## 2. Results

### 2.1. Role of 6-Hydroxydopamine (6-OHDA) in Decreased Cell Viability of SH-SY5Y Cells

**Figure 2:**
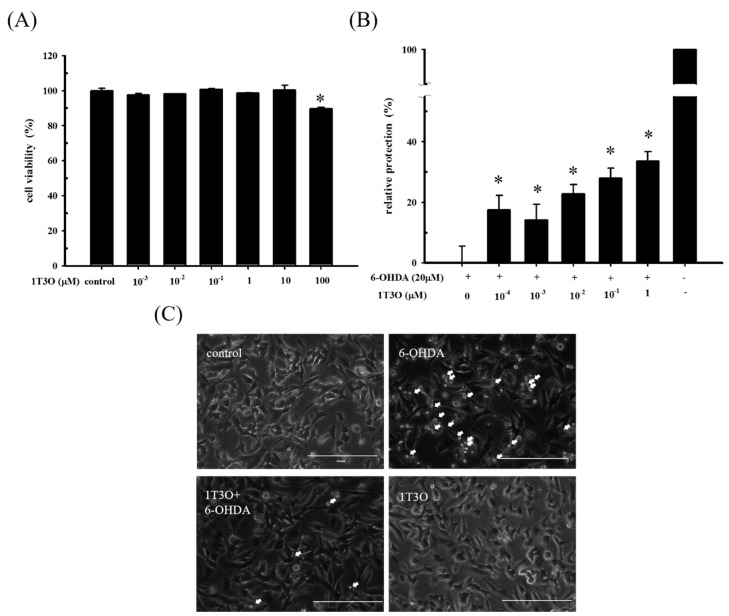
The neuroprotective effect of 1T3O on 6-OHDA-induced neurotoxicity in SH-SY5Y cells. SH-SY5Y cells were preincubated for 1 h with the indicated concentrations of 1T3O and then challenged with 6-OHDA (20 μM). (**A**) effect on SH-SY5Y cell viability after 16 h 1T3O treatment at different concentrations (0, 100, 10, 1, 10^−1^, 10^−2^, and 10^−3^ μM) is shown. Data were represented by mean ± SEM. (* *p* < 0.05 vs. Control); (**B**) relative protection of control (without the treatment of 6-OHDA and 1T3O) and 6-OHDA-treated alone group were taken to be 100% and 0%, respectively. This experiment was repeated for six times. *, significantly different from the 6-OHDA-treated alone group (*p* < 0.05); (**C**) an inverted optical microscope was used to study the morphological changes in 6-OHDA-treated SH-SY5Y cells induced by 1T3O. Apoptotic cells were indicated by arrows. 1 μM; scale bar = 100 μm.

To determine a suiTable 1T3O concentration for subsequent experiments, an alamarBlue^TM^ reduction assay was used to study SH-SY5Y cell viability after 16 h of treatment with different concentrations of 1T3O (0, 10^−3^, 10^−2^, 10^−1^, 1, 10, 100 μM). Results show that cell viability was not affected by 1T3O concentrations in the range of 10^−3^ to 10 μM. When the treatment concentration exceeded 100 μM, a significant reduction in cell viability was observed, compared with the control (Figure 2A). SH-SY5Y cell viability was measured after a 1 h pretreatment with 1T3O (10^−4^, 10^−3^, 10^−2^, 10^−1^, 1 μM) and subsequently challenged with 6-OHDA (20 μM) for 16 h. Results show that in the concentration range of 10^−4^ to 1 μM, 1T3O demonstrated a neuroprotective effect and effectively inhibited 6-OHDA-induced reduction in cell viability (Figure 2B). Characteristic morphological changes associated with apoptosis, such as significant shrinkage, deformation, etc., were observed in SH-SY5Y cells treated with 6-OHDA (20 μM) for 8 h (Figure 2C). After pretreatment with 1T3O (1 μM), 6-OHDA-induced apoptosis was attenuated (Figure 2C). Results showed that 1T3O effectively inhibited 6-OHDA-induced cell apoptosis and subsequent experiments were conducted at the concentration with maximum cell protection of 1 μM.

### 2.2. Effect of 1T3O on 6-OHDA-Induced SH-SY5Y Neuroblastoma Apoptosis

**Figure 3:**
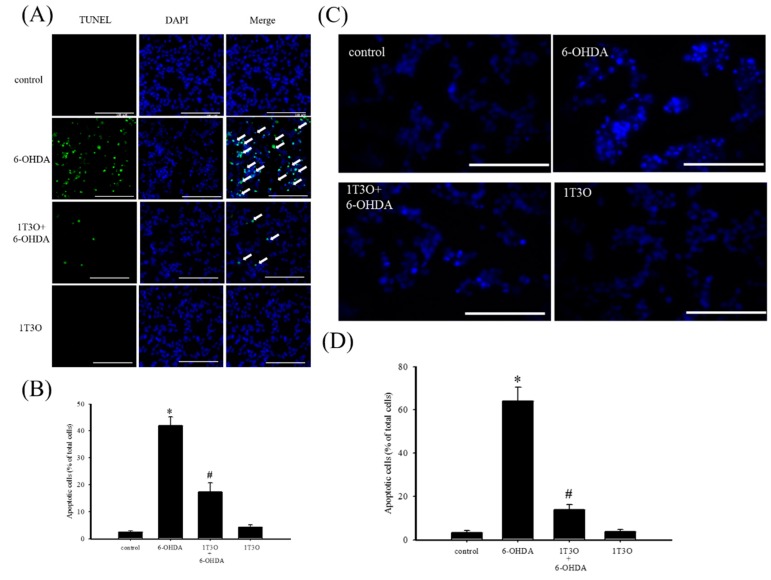
The effect of 1T3O on 6-OHDA-induced apoptosis in SH-SY5Y cells. (**A**) TUNEL stain was used to study the effect of 1T3O on 6-OHDA-induced apoptosis in SH-SY5Y cells. The TUNEL signal is shown in fluorescent green and the stained nuclei are shown in fluorescent blue (4′,6-diamidino-2-phenylindole, DAPI). Apoptotic cells were indicated by arrows. (**B**) represents the quantitative analysis result of the TUNEL stain signal; (**C**) shows the use of Hoechst stain to study the effect of 1T3O on 6-OHDA-induced apoptosis in SH-SY5Y cells. Blue fluorescence is the Hoechst stain signal; (**D**) represents the quantitative analysis result of Hoechst stain signal. Results show that 1T3O significantly inhibited 6-OHDA-induced cell toxicity. Data are represented by mean ± SEM. (* *p* < 0.05 vs. Control; ^#^ *p* < 0.05 vs. 6-OHDA group; scale bar = 100 μm).

TUNEL stain was used to study cell apoptosis. Cell apoptosis causes DNA fragmentation and DNA fragments can be identified by terminal deoxynucleotidyl transferase, which catalyzes 2′-deoxyuridine 5′-triphosphate (d-UTP) incorporation on the 3′-OH end of DNA, producing fluorescence. Results show that, compared with the control group (Figure 3A), a greater fluorescent signal, corresponding to a higher degree of chromosomal DNA fragmentation associated with cell apoptosis, was noted in neuroblastoma cells treated with 6-OHDA (20 μM) for 8 h (Figure 3A). On the other hand, 6-OHDA-induced fluorescent signal decreased with 1T3O (1 μM) pretreatment (Figure 3A). Quantitative analysis found that 1T3O can effectively inhibit 6-OHDA-induced cell apoptosis. Furthermore, Hoechst stain can also be used to study cell apoptosis. Because cell apoptosis causes an increase in membrane permeability and changes in chromosome structure, Hoechst stain can penetrate the cell membrane and bind to the DNA to allow fluorescent labeling. The Hoechst stain binds to the chromosomal DNA of apoptotic cells and emits fluorescence. Compared with the control group (Figure 3C), 15 h of treatment with 6-OHDA (20 μM) resulted in a stronger apoptosis-indicating fluorescent signal. On the other hand, pretreatment with 1T3O (1 μM) resulted in a reduction in 6-OHDA-induced fluorescent signal (Figure 3C). Quantification analysis shows that 1T3O can significantly inhibit 6-OHDA-induced cell apoptosis (Figure 3D). 

### 2.3. Effect of 1T3O on the Akt (Phospho-Akt), Phospho-Extracellular Signal-Regulated Kinase (Phospho-ERK), and Phospho-p38 Protein Expression in SH-SY5Y Cells

**Figure 4:**
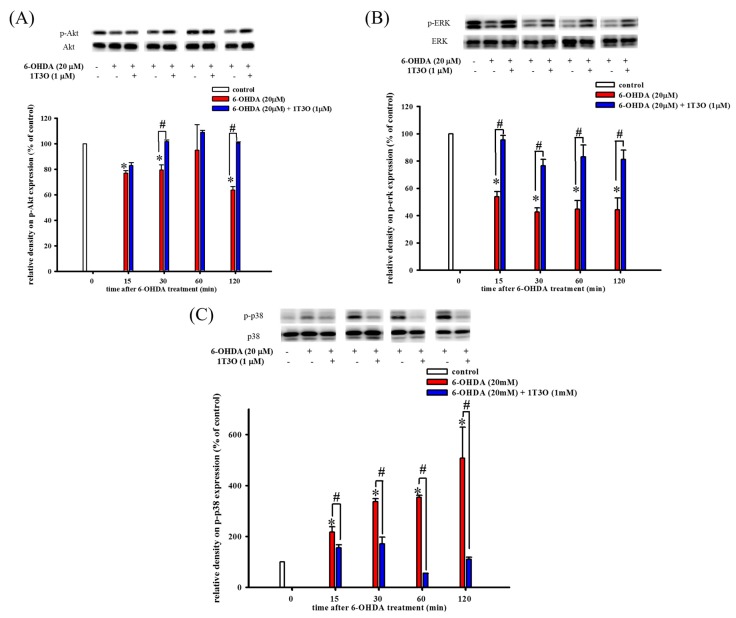
Effect of 1T3O on 6-OHDA-induced reduction in protein kinase B (Akt) phosphorylation, extracellular signal-regulated kinase (ERK) phosphorylation, and increase in p38 phosphorylation. Western blot was used to study the expression of phosphorylated Akt (phospho-Akt), phosphorylated ERK (phospho-ERK) and phosphorylated p38 (phospho-p38). The effect of 1T3O (1 μM) on 6-OHDA-induced (20 μM) reduction in Akt phosphorylation (**A**), ERK phosphorylation (**B**), and increase in p38 phosphorylation (**C**) at different time points was analyzed. Data are presented as mean ± SEM. (* *p* < 0.05 vs. Control; ^#^ *p* < 0.05 vs. 6-OHDA group of the same time point).

Western blot was used to study the effect of 6-OHDA on the expression of phospho-Akt (Figure 4A), phospho-ERK (Figure 4B), and phospho-p38 (Figure 4C) in cells. The PI3K/Akt signaling pathway plays an important role in maintaining cell viability. Results show that, compared with the control, 15 min of treatment with 6-OHDA (20 μM) significantly decreased the expression of phosphorylated Akt (Figure 4A). On the other hand, pretreatment with 1T3O (1 μM) for 30 min significantly attenuated 6-OHDA (20 μM)-induced reduction in phosphorylated Akt level (Figure 4A). Thus, these results demonstrate that 1T3O can significantly inhibit 6-OHDA-induced reduction in Akt phosphorylation. Although ERK and p38 are both members of the MAPK protein family, their functions differ significantly. The ERK signal transduction pathway regulates mitosis, cell growth, and inhibits apoptosis. Results show that, compared with the control, 15 min of treatment with 6-OHDA (20 μM) resulted in a significant reduction in ERK expression (Figure 4B), and pretreatment with 1T3O (1 μM) resulted in the inhibition of the reduction of phosphorylated ERK after 15 min (Figure 4B). On the other hand, p38 phosphorylation results in the downstream activation of relevant proteins in the apoptosis pathway, leading to cell death. Results show that, compared with the control, treatment with 6-OHDA (20 μM), for 15 min significantly increased the expression of phosphorylated p38 (Figure 4C), whereas pretreatment with 1T3O (1 μM) for 15 min significantly inhibited 6-OHDA-induced increase in p38 phosphorylation (Figure 4C). Taken together, results showed that 1T3O can significantly inhibit 6-OHDA-induced reduction in ERK phosphorylation and increase in p38 phosphorylation. On the other hand, 1T3O treatment alone did not affect the expression of phospho-ERK and phospho-p38.

### 2.4. Effect of 1T3O on 6-OHDA-Induced Activation of Cellular Caspase-3

**Figure 5:**
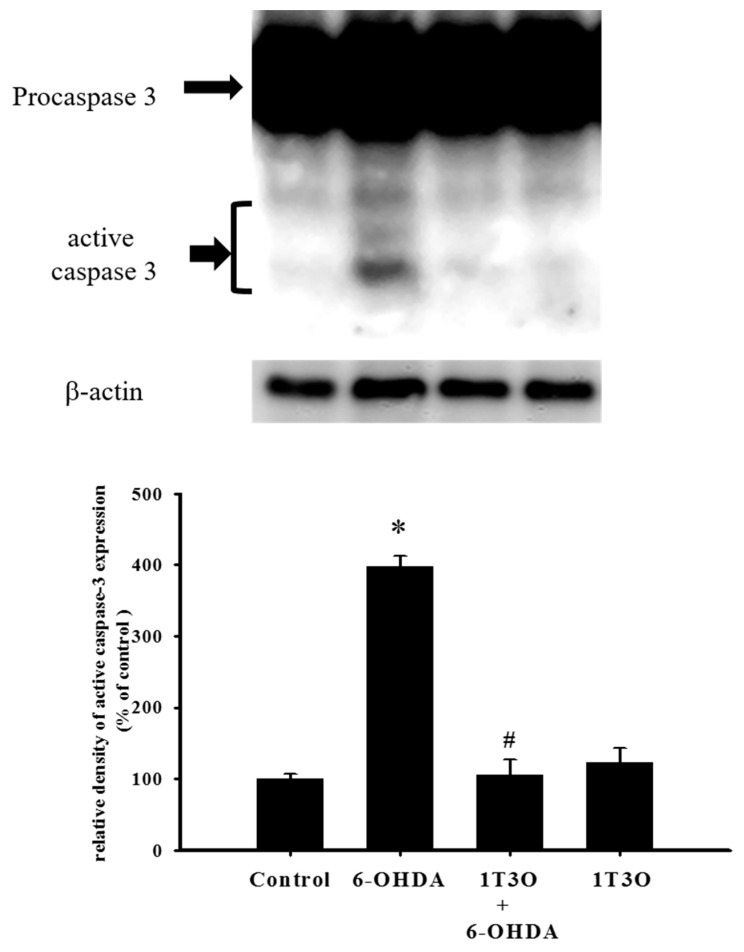
Effect of 1T3O on 6-OHDA-induced caspase-3 activation. Western blot was used to analyze the expression of caspase-3 and study the effect of 1T3O (1 μM) on 6-OHDA-induced (20 μM) caspase-3 activation. 1T3O significantly attenuated 6-OHDA induced caspase-3 activation. Data are represented by mean ± SEM. (* *p* < 0.05 vs. Control; ^#^ *p* < 0.05 vs. 6-OHDA group).

Caspase-3 is a key protein mediating apoptosis. Activation of caspase-3 triggers apoptosis. Results showed that, compared with the control group, treatment with 6-OHDA (20 μM) significantly increased the expression of activated (cleaved) caspase-3 (Figure 5). Pretreatment with 1T3O (1 μM) significantly attenuated 6-OHDA-induced increase in the expression level of activated caspase-3 (Figure 5). Therefore, results demonstrated that 1T3O can significantly inhibit 6-OHDA-induced caspase-3 activation.

### 2.5. Effect of 1T3O on 6-OHD-Induced Increase in Oxidative Stress in SH-SY5Y Cells

**Figure 6:**
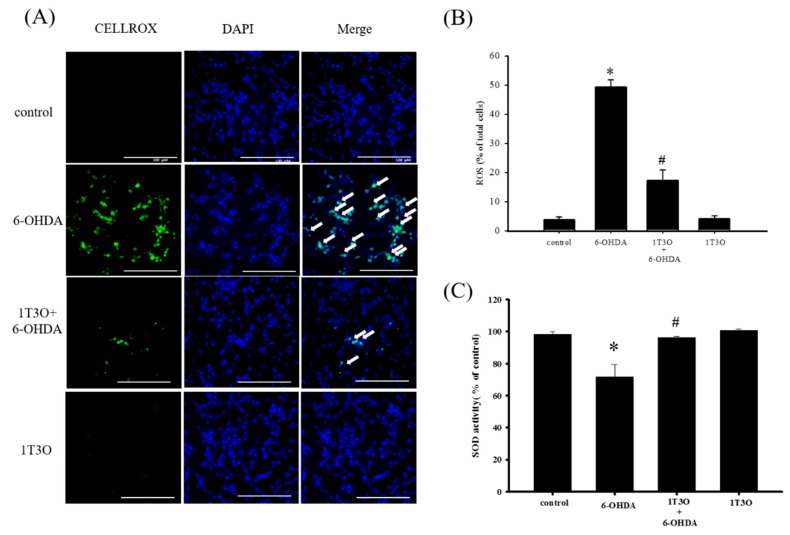
Effect of 1T3O on 6-OHDA-induced elevation in cellular ROS and decrease in superoxidase dismutase (SOD) activity. (**A**) Shows the use of CellROX^®^ stain as a specific fluorescent probe for intracellular ROS to detect intracellular oxidative stress. Green fluorescence represents bounded CellROX^®^ stain and blue fluorescence represents DAPI-dyed cell nuclei. Arrows indicate the ROS generated in cells. ROS level was shown as (**B**); (**C**) represents the use of the SOD activity assay kit to quantitate intracellular SOD activity and study the effect of 1T3O (1 μM) on 6-OHDA-induced (20 μM) reduction in SOD activity. Results show that 1T3O can attenuate 6-OHDA-induced ROS elevation and SOD activity reduction. Data are presented as mean ± SEM. (* *p* < 0.05 vs. Control; ^#^ *p* < 0.05 vs. 6-OHDA group).

A significant amount of ROS was generated in SH-SY5Y cells after treatment with 6-OHDA. This study used the CellROX^®^ stain to specifically bind ROS inside cells to detect intracellular oxidative stress. Results showed that, compared with the control, treatment with 6-OHDA (20 μM) generated a significant amount of ROS, and pretreatment with 1T3O (1 μM) inhibited 6-OHDA-induced ROS elevation. Treatment with 1T3O (1 μM) alone did not produce any fluorescent signals (Figure 6A). Quantification analysis (Figure 6B) shows that 1T3O can significantly inhibit 6-OHDA-induced ROS elevation. On the other hand, SOD is an important antioxidant enzyme in the body and is capable of catalyzing the dismutation of superoxide into oxygen or oxygen peroxide. In this study, SOD activity was used to represent the antioxidant ability of cells. Results showed that, compared with the control group, 6-OHDA (20 μM) treatment significantly decreased SOD activity (Figure 6C). On the other hand, pretreatment with 1-μM 1T3O significantly attenuated 6-OHDA-induced reduction in SOD activity (Figure 6C). In summary, results demonstrated that pretreatment with 1T3O can significantly inhibit 6-OHDA-induced elevation in oxidative stress.

### 2.6. Effect of 1T3O on 6-OHDA-Induced Changes in Proteins Related to the Akt/Nrf2 Signaling Pathway of SH-SY5Y Neuroblastomas

**Figure 7:**
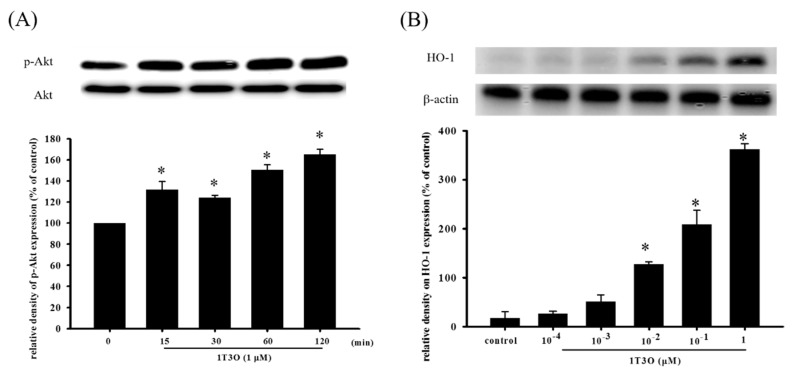
The effect of 1T3O on phospho-Akt and heme oxygenase-1 (HO-1) protein levels in SH-SY5Y cells. Western blot was used to quantitate phospho-Akt and HO-1 protein levels and the effect of 1T3O (1 μM) on Akt phosphorylation at different time points (**A**) and the effect of different 1T3O concentrations (1, 10^−1^, 10^−2^, 10^−3^, and 10^−4^ μM) on HO-1 protein level (**B**) were studied. 1T3O significantly increased the protein expression of phospho-Akt and HO-1. Data are presented as mean ± SEM. * *p* < 0.05 vs. Control.

**Figure 8:**
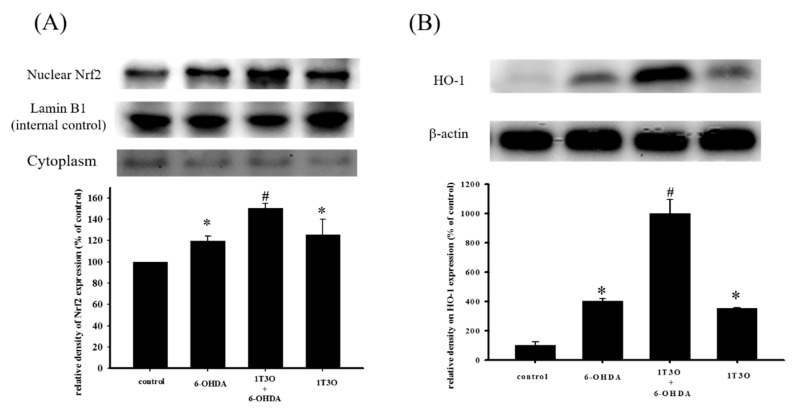
Effect of 1T3O on 6-OHDA-induced increase in intra-nuclear transcription factor Nrf2 and HO-1 expression. Western blot was used to quantitate the expression of intra-nuclear Nrf2 and HO-1, and the effect of 1T3O (1 μM) on 6-OHDA (20 μM)-induced increase in Nrf2 (**A**) and HO-1 (**B**) expression. 1T3O significantly increased 6-OHDA-induced elevation in intra-nuclear Nrf2 and HO-1 expression levels. Data were presented as mean ± SEM. (* *p* < 0.05 vs. Control; ^#^ *p* < 0.05 vs. 6-OHDA group)

Studies have shown that Akt participates in regulating Nrf2 nuclear translocation and affects the expression of the downstream HO-1 protein, an important antioxidative stress protein. Results show that treatment with 1T3O (1 μM) alone for 15, 30, 60, and 120 min significantly increased the expression level of phosphorylated Akt (phospho-Akt; Figure 7A). On the other hand, 1T3O treatment (10^−2^, 10^−1^, 1 μM) alone for 24 h (Figure 7B) significantly increased HO-1 expression in SH-SY5Y cells. 1T3O concentrations in the range of 10^−3^ to 10^−4^ μM did not have any effect on HO-1 expression. Results showed that compared with the control group, treatment with 6-OHDA (20 μM) resulted in significant elevation in intra-nuclear Nrf2 (Figure 8A) and HO-1 (Figure 8B) levels. Pretreatment of cells with 1T3O (1 μM) resulted in further increases in 6-OHDA-induced increases in intra-nuclear Nrf2 (Figure 8A) and HO-1 (Figure 8B).

### 2.7. Attenuation of the Neuroprotective Effect of 1T3O by LY294002 and Zinc Protoporphyrin (ZnPP)

**Figure 9:**
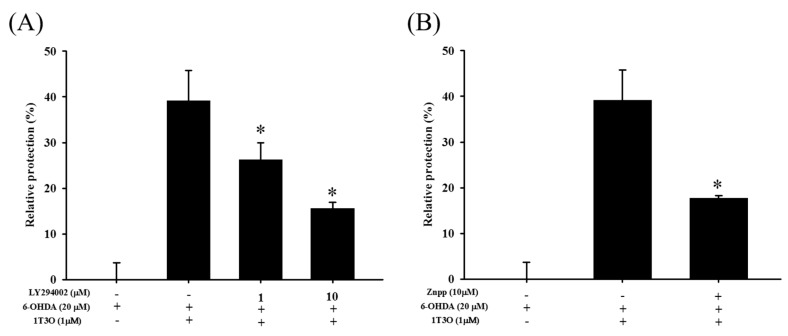
Effect of LY294002 and ZnPP on 6-OHDA-induced SH-SY5Y cell death inhibition by 1T3O. The alamarBlue^TM^ reduction assay was used to study the effect of LY294002 (1, 10 μM), a PI3K inhibitor, and Zinc Protoporphyrin (ZnPP) (10 μM), an HO-1 inhibitor, on 1T3O inhibition of 6-OHDA-induced decrease in cell viability. Relative protection was calculated. Data are presented as mean ± SEM. * *p* < 0.05 vs. 6-OHDA group with 1 h 1T3O pretreatment.

LY294002 is a specific inhibitor of PI3K. Results show that LY294002 treatment (1 and 10 μM) significantly inhibited the neuroprotective effect of 1T3O in 6-OHDA-induced SH-SY5Y cell death (Figure 9A). On the other hand, zinc protoporphyrin (ZnPP) is a specific inhibitor of HO-1. Results show that ZnPP treatment (10 μM) significantly inhibited the neuroprotective effect of 1T3O in 6-OHDA-induced SH-SY5Y cell death (Figure 9B).

### 2.8. Effect of 1T3O on Zebrafish Mortality

To determine suitable 1T3O concentration for use in subsequent zebrafish experiments, zebrafish embryos were treated with different concentrations of 1T3O (10^2^, 50, 5, 5 × 10^−1^, 5 × 10^−2^, and 5 × 10^−3^ μM) 9 h to four days after fertilization; and zebrafish mortality was recorded four days after fertilization. Results show that treatment with 1T3O at a concentration of 50 μM did not result in significant mortality, whereas treatment at a concentration of 100 μM resulted in zebrafish mortality (Table 1).

### 2.9. Effect of 1T3O on 6-OHDA-Induced Deficits in Zebrafish Locomotor Activity

**Figure 10:**
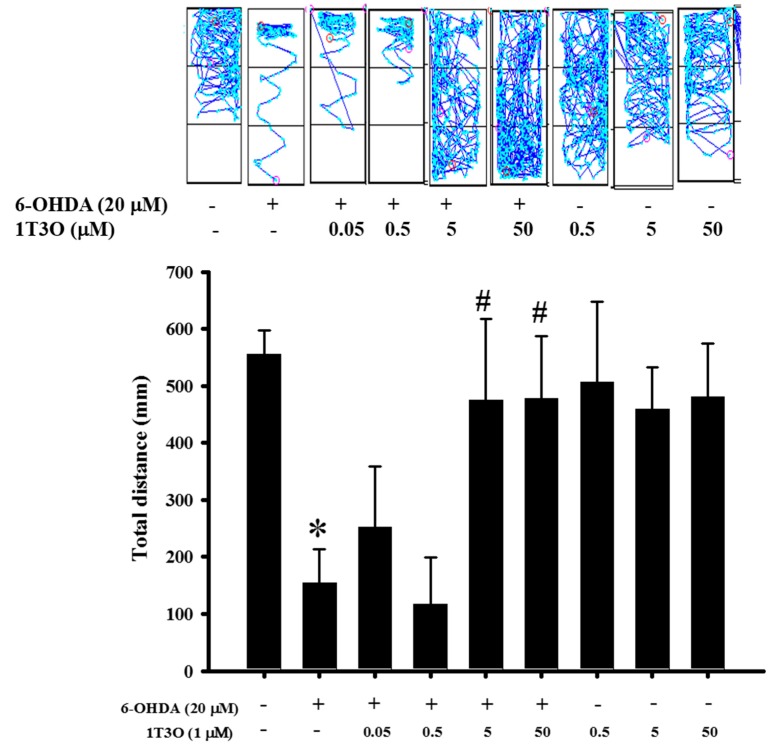
Effect of 1T3O on 6-OHDA-induced deficits in locomotor activity of zebrafish. The figure represents the effect of 1T3O on 6-OHDA-induced deficits in zebrafish locomotor activity. Results show that 1T3O can effectively ameliorate 6-OHDA-induced deficits in zebrafish locomotor activity. Data are presented as mean ± SEM. Total number of animals = 12; * *p* < 0.05 vs. Control group without any treatment; ^#^ *p* < 0.05 vs. 6-OHDA-only group.

Previous results verified the neuroprotective activity of 1T3O in an in vitro model. Thus, an in vivo experiment was conducted to further confirm this effect. Results show that the total swimming distance of zebrafish in the 6-OHDA (250 μM) group was significantly lower than that in the control group. On the other hand, the pretreatment of zebrafish embryos with different concentrations of 1T3O (5 and 50 μM) ameliorated the 6-OHDA-induced reduction in total swimming distance. No significant effects were observed after treatment with 1T3O at 5 × 10^−1^-μM, 5 × 10^−2^-μM, and 5 × 10^−3^-μM concentrations (Figure 10). In summary, these results show that 1T3O (5 and 50 μM) can effectively attenuate 6-OHDA-induced deficits in locomotor activity.

### 2.10. Effect of 1T3O on 6-OHDA-Induced Changes in the Expression Levels of TNF-α and TH in Zebrafish Brain Tissue

**Figure 11:**
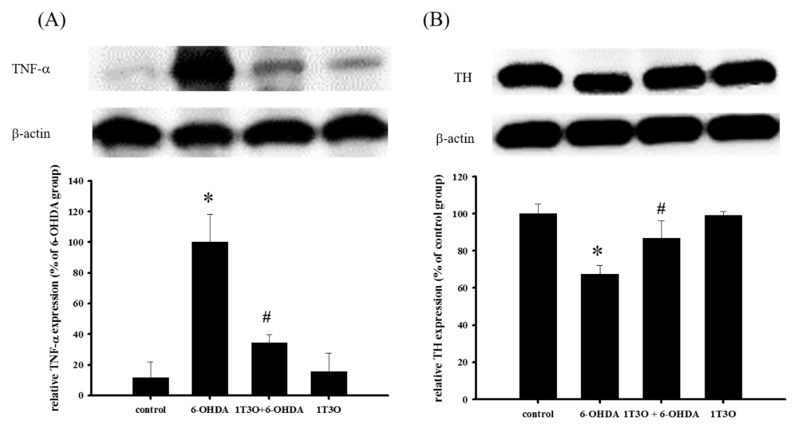
Effect of 1T3O on 6-OHDA-induced increase in inflammatory factor TNF-α expression and decrease in tyrosine hydroxylase (TH) expression in zebrafish brain tissue. Western blot was used to analyze expression of TNF-α and TH protein, and to study the effect of 1T3O on 6-OHDA-induced elevation in TNF-α expression and reduction in TH expression. Results showed that 1T3O can ameliorate 6-OHDA-induced increase in TNF-α and decrease in TH. Data are presented as mean ± SEM. Total number of animals = 12; * *p* < 0.05 vs. Control; ^#^ *p* < 0.05 vs. 6-OHDA group.

TNF-α is a proinflammatory protein and is considered to be an inflammatory marker. Results showed that, compared with the control, treatment with 6-OHDA (250 μM) increased the expression of TNF-α. On the other hand, pretreatment with 1T3O (5 μM) significantly inhibited 6-OHDA-induced increase in zebrafish TNF-α (Figure 11A). TH is a marker for dopaminergic neurons. Results showed that, compared with the control, 6-OHDA (250 μM) treatment resulted in the significant reduction of TH expression. On the other hand, pretreatment with 1T3O (5 μM) significantly inhibited TH reduction in zebrafish (Figure 11B). Results showed that 1T3O can significantly inhibit 6-OHDA-induced elevation of TNF-α and effectively restore TH expression that was decreased in zebrafish brains by 6-OHDA.

## 3. Discussion

### 3.1. Compounds Conferring Neuroprotection via the Nrf/HO-1 Pathway

The Nrf2/HO-1 pathway is one of the important pathways protecting against oxidative stress in the body; wherein Nrf2, the most well-known antioxidant response element (ARE)-associated transcription factor, enters the cell nucleus on activation and binds to AREs to initiate transcription [18], inducing the production of downstream antioxidant proteins such as HO-1, SOD, NAD(P)H:quinone-oxidoreductase-1 (NQO1), lactaldehyde reductase (NAD(P)H), and glutathione peroxidase (GSHP) [19]. A separate study demonstrated that MAPKs and PI3K/Akt can promote the entry of nuclear factor erythroid 2–related factor 2 (Nrf2) into the nucleus and increase the expression of HO-1, thereby protecting the cell from oxidative stress [41]. Studies showed that HO-1 is an important intracellular protein that protects against external stimulus and oxidative stress [42,43,44]. HO-1 is a rate-limiting enzyme in heme catabolism that is present mainly in the endoplasmic reticulum and interacts with the NADPH cytochrome P450 reductase to convert heme to ferrous ion (Fe^2+^), carbon monoxide (CO), and biliverdin [42]. Biliverdin is reduced by biliverdin reductase to bilirubin, an important antioxidant compound in hemoglobin [45,46]. Previous studies have demonstrated the effect of bilirubin in inhibiting cellular apoptosis [47,48,49]. Heme, on the other hand, is itself a strong prooxidant. Activated HO-1 metabolizes heme and decreases oxidative stress within the cell [50]. Thus, HO-1 is believed to have antioxidant, antiinflammatory, and antiapoptotic effects. HO-1 expression and activation is a critical mechanism that protects cells and tissues from external stress and stimulus. Because oxidative stress, neuroinflammation, and apoptosis of dopaminergic neurons play significant roles in PD pathogenesis, regulation of the above mechanisms to achieve neuroprotection may most likely lead to the postponement or even treatment of PD. A study demonstrated that the clinical treatment drug deprenyl can partially regulate the Nrf2 transcription factor to achieve a neuroprotective effect [26]. Deprenyl is a type of monoamine oxidase B (MAO-B) inhibitor that has been shown to show an antioxidant effect in the Nrf2/HO-1 pathway [26,51]. These results show that deprenyl can activate the PI3K and MAPK pathways to significantly activate Nrf2 to produce NQO1 and HO-1, protecting the cell from oxidative stress [26,51]. Other studies showed that deprenyl treatment effectively restores 1-methyl-4-phenyl-1,2,3,6-tetrahydropyridine-induced (MPTP-induced) deficits in locomotor activity in a rat PD model, and restore the population of substantia nigra dopamine neurons in comparison with the pathological group [52,53]. However, clinical results showed that this drug cannot fully cure PD and induces side effects such as insomnia, hallucination, delusions, among others. On the other hand, butein is a clinical drug with high antioxidant ability that is currently used for the treatment of breast cancer and colorectal cancer. However, because of its excellent antioxidant ability, it is also being studied for clinical use in PD treatment [54,55,56]. Moreover, other potential neuroprotective compounds, such as baicalein and astaxanthin, have also been studied for their effects on the Nrf2/HO-1 pathway. A previous study showed that treatment with 6-OHDA activates the intracellular endogenous antioxidant mechanism, and promotes Nrf2 activation and its entry into the cell nucleus to form a complex with ARE sequences to initiate transcription, producing downstream HO-1 which counters oxidative stress and protects the cell [57]. On the other hand, in addition to its ability to ameliorate 6-OHDA-induced apoptosis and oxidative stress elevation, pretreatment with baicalein (200 μM) also increases Nrf2 expression compared with the 6-OHDA group, confirming that baicalein can antagonize 6-OHDA-induced oxidative stress via the activation of Nrf2, thereby preventing apoptosis [57]. Astaxanthin is a well-known antioxidant and a previous study has shown that astaxanthin can significantly inhibit 1-methyl-4-phenylpyridinium -induced (MPP^+^-induced) oxidative stress and apoptosis in PC12 cells [58]. Furthermore, its role in affecting relevant protein expression in the Nrf2 pathway is similar to what was found in this study; MPP^+^ causes increased Nrf2 and HO-1 expression and co-treatment of MPP^+^ with astaxanthin further increased Nrf2 and HO-1 expression. The above mentioned studies all verify that the working mechanism of PD-antagonists is closely associated with the activation of the Nrf2/HO-1 pathway. This study showed that treatment with 1T3O alone at different concentrations for 24 h significantly increased the amount of HO-1, compared with the control group (Figure 7B). Consistent with the findings of the previous study, Nrf2 and HO-1 expression in the 6-OHDA alone group were significantly higher than those in the control group; and pretreatment with 1T3O augmented the 6-OHDA-induced increase in Nrf2 (Figure 8A) and overall HO-1 (Figure 8B) expression. This study utilized the ability of 6-OHDA to produce large quantities of superoxides and ROS, combined with the specificity of the fluorescent probe CellROX^®^ stain, which binds intracellular ROS and is a method for detecting SOD activity, to study intracellular oxidative stress. Results verified that 1T3O treatment can inhibit 6-OHDA-induced intracellular oxidative stress (Figure 6A,B) and restore SOD activity (Figure 6C). Furthermore, it is worth mentioning that 1T3O treatment alone significantly increased SOD viability, compared with the control group (Figure 6C). A study showed that SOD and HO-1 are both inducible proteins [59]. On the other hand, this study found that 1T3O itself could regulate the endogenous antioxidant mechanism. In comparison with previous studies, where epigallocatechin gallate (EGCG) (10–100 μM) [60], astaxanthin (20 μM) [58], and baicalein (200 μM) [57] were used, the concentration of 1T3O used in this study (1 μM) was significantly lower than the concentrations of other drugs. Moreover, treatment with 1T3O alone produced significantly higher Nrf2 expression than the control group. Compared with the 6-OHDA group, 1T3O treatment further significantly increased Nrf2 expression (Figure 8A). To verify that the neuroprotective effect of 1T3O is through the Nrf2/HO-1 pathway, this study used a specific HO-1 inhibitor, ZnPP. Results showed that the addition of 10 μM of ZnPP significantly inhibited the neuroprotective effect of 1T3O (Figure 9B). This result further confirmed that the neuroprotective mechanisms of 1T3O are because of the activation of Nrf2/HO-1 pathway and attenuation of oxidative stress.

### 3.2. Relevant Compounds Inhibiting the p38 Pathway and Activating the ERK Pathway

Regulation of p38 and ERK, members of the MAPK family, is closely related to cellular apoptosis and growth. Currently, clinical drugs and neuroprotective compounds on the market, such as ranitidine [61] and loganin [62], exert a neuroprotective effect by regulating p38 and ERK. A previous clinical study found significantly higher amounts of histamine in the blood of PD patients compared with healthy individuals [63]. This led to a further study on the neuroprotective role of H2 receptor antagonists. That study showed that co-treatment with ranitidine effectively protects SH-SY5Y cells from damage caused by rotenone [61], and this mechanism involves the activation of p-ERK and its downstream antiapoptotic protein, Bcl-1, thus inhibiting the production of rotenone-induced p38 and downstream caspase-3, to exert a protective effect. This result revealed that p-ERK and p-p38 are potential targets for neuroprotection [61]. Another study showed that the potential compound loganin has a neuroprotective effect in vitro. Results showed that loganin treatment can effectively inhibit H_2_O_2_-induced SH-SY5Y apoptosis and the protection mechanism is attributed to decreased apoptosis as a result of p38, caspase-3, and cytochrome c regulation. Additionally, loganin treatment increases p-ERK expression, causing downstream Bcl-2 expression to increase, attenuating cell apoptosis [62]. Although clinical drug levodopa is the most commonly prescribed drug for PD, previous studies had clearly showed that levodopa administration increases p38 expression in SH-SY5Y cells and further activates downstream caspase-3. Results showed that although levodopa can effectively improve clinical symptoms of PD, it can also accelerate the course of the disease at the same time [64]. Therefore, the development of new drugs is of great importance. 1T3O, used in this study in place of levodopa, inhibited 6-OHDA-induced p38 activation and p-ERK reduction, acting showing similar effects as loganin and ranitidine.

### 3.3. Inhibiting 6-OHDA-Induced SH-SY5Y Apoptosis via the Akt Pathway

Several studies have demonstrated the neuroprotective effect of potential neuroprotective compounds, including epigallocatechin gallate (EGCG) [60] and resveratrol [65], etc., in an in vitro PD model. Epigallocatechin gallate is the most abundant flavonoid in tea leaves and has been shown to show antioxidant and free radical scavenging effects [66]. The administration of EGCG significantly activates the PI3K/Akt pathway, thereby inhibiting 6-OHDA-induced SH-SY5Y apoptosis [60]. Another study showed that, in a *rat* PD model, the administration of EGCG effectively prevented significant increases in ROS and CO that were induced by 6-OHDA and can moderate the loss of dopamine neurons [67]. On the other hand, resveratrol is a compound present in the skin of red grapes and wine, and is used widely in studies on inhibiting cancer cell growth and preventing cardiovascular and NDs [68]. A study using oxyresveratrol, a resveratrol derivative, showed that oxyresveratrol (10 μM) shows excellent neuroprotective activity and can effectively inhibit neurotoxin 6-OHDA-induced apoptosis. At 10 μM, oxyresveratrol also attenuates 6-OHDA-induced abnormal cell morphology and upregulates the PI3K/Akt pathway [65]. The same study attributed the neuroprotective effect of oxyresveratrol to regulation of proteins in the PI3K pathway. This study used 1T3O, a neuroprotectant with similar effects as ECCG and oxyresveratrol, which not only inhibited 6-OHDA-induced apoptosis (Figure 8B) and changes in cell morphology (Figure 3C), but also restored the expression of Akt (Figure 4A). Furthermore, the neuroprotective effect of 1-μM 1T3O is comparable to 10-μM oxyresveratrol, demonstrating that 1T3O has an added advantage of a lower effective dose, which is a significant advantage in the future development of the compound into a clinical drug. This study not only directly observed the changes in relevant proteins in the PI3K pathway after 1T3O treatment but also used the PI3K pathway inhibitor, LY294002, to confirm inversely the role of this pathway in the therapeutic effect of 1T3O. Results showed that, after LY294002 administration, the restoration of 6-OHDA-induced apoptosis by 1T3O was significantly inhibited (Figure 9A). Although this study was not conducted in a *rat* model, an in vivo zebrafish PD model was used and demonstrated the neuroprotective effect of 1T3O. Therefore, we hypothesized that the neuroprotective effect of 1T3O may be related to the activation of the PI3K/Akt signaling pathway, as well as activation of HO-1 to protect cells from damage caused by oxidative stress.

### 3.4. Analysis of the Neuroprotective Effect of the Compound in a Zebrafish PD Model

Tremors, rigidity, bradykinesia, and other clinical symptoms of PD are not easily observed in common PD animal models used in drug development such as *Drosophila*, *zebrafish*, and *rat* [69]. Therefore, current in vivo studies mostly adopt locomotor activity to determine the severity of PD. In a zebrafish model, the slowing of movement is similar to the clinical features of PD in humans [70]. Mammalian PD models, however, require the use of amphetamine to induce rotational behavior. A previous study used a zebrafish model to investigate the changes in behavior [71] after administration of 6-OHDA and the compound of interest to analyze the neuroprotective activity of the compound. Similarly, this study used 6-OHDA to induce damage in dopaminergic neurons in zebrafish larvae and used a software to record and analyze the typical path and swimming distance of zebrafish larvae in real-time. Our team also used this zebrafish imaging system to evaluate several clinical drugs such as a-tocopherol, minocycline, Sinemet, and others, and confirmed the relationship between this model and inflammation, oxidative stress, and death of dopaminergic neurons, through analysis of molecular markers such as TH, THF-α, pink1, and parkin [72]. This study also verified the feasibility and practicality of the zebrafish model. Results of this study showed that, compared with the control group, administration of 6-OHDA resulted in significant reduction in total swimming distance and pretreatment with 1T3O improved 6-OHDA-induced reduction in total swimming distance (Figure 10).

### 3.5. Neuroprotective Mechanism of the Compound in the Zebrafish PD Model

**Figure 12:**
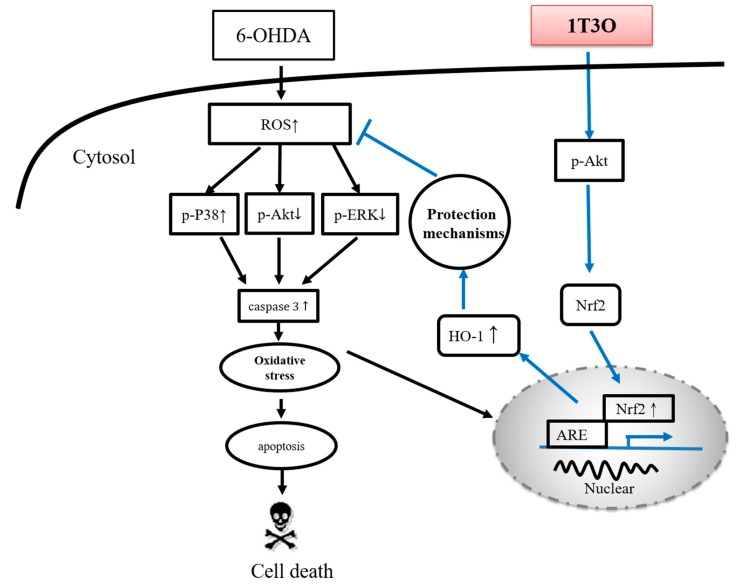
Neuroprotective mechanism of 1T3O. When cells are stimulated by 6-OHDA, a significant increase in ROS is observed. The transcription factor Nrf2 is activated, enters the nucleus, and binds to AREs to initiate transcription and induce the production of type-II enzymes, HO-1 and SOD, for cell protection. This is known as the endogenous antioxidant mechanism. 6-OHDA causes cellular apoptosis by decreasing the expression of phosphorylated Akt and ERK, and significantly activating p38 phosphorylation, resulting in caspase-3 activation. Administration of 1T3O activates the PI3K/Akt signaling pathway and causes the transcription factor Nrf2 to enter the nucleus and bind to AREs to induce an increase in both HO-1 expression and SOD activity to protect cells and counter oxidative stress. In comparison with the administration of 6-OHDA alone, the administration of 1T3O can better activate this signaling pathway to elicit its protective effects. In addition, 1T3O can inhibit the expression of caspase-3 via the phosphorylation of Akt and ERK, and can also attenuate the activation of p38 to prevent subsequent activation of caspase-3 expression to achieve its antiapoptotic and neuroprotective effects.

The main pathological feature of PD is the loss of dopaminergic neurons in the substantia nigra par compacta [73]. According to studies, tyrosine is the precursor of dopamine and is catalyzed by a series of different enzymes to produce dopamine [74]; wherein, TH is a key enzyme as well as the rate-limiting enzyme that catalyzes tyrosine to l-dihydroxyphenylalanine before it is further converted to dopamine [75]. Therefore, tyrosine hydroxylase (TH) is considered the marker of dopaminergic neurons in various PD studies [75,76,77]. Amantadine is a clinical drug currently prescribed for the treatment of PD and demonstrates an antiinflammatory effect. According to a previous study, amantadine can effectively inhibit the release of proinflammatory cytokines, prostaglandin E2 (PGE2), and TNF-α, caused by LPS-induced or 6-OHDA-induced microglia activation in a rat PD model [78]. Moreover, a separate study showed that amantadine can effectively improve MPTP-induced increase in rotational behavior in an MPTP mouse model of PD, and the number of dopaminergic neurons observed was also greater than that noted in the MPTP group [79]. In this study, samples were collected from zebrafish brains and Western blot was used to detect TH expression. Results showed that the administration of 1T3O 9 h to 4 days after fertilization, followed by administration of 6-OHDA 2–4 days after fertilization resulted in improvement of 6-OHDA-induced reduction in TH protein expression in zebrafish brains (Figure 11B). On the other hand, 1T3O also significantly inhibited neurotoxin 6-OHDA-induced elevation in proinflammatory cytokine TNF-α (Figure 11A). The above results demonstrated that 1T3O may have a similar treatment mechanism as current clinical drugs and has the potential for further development. According to the above results, the 1T3O-associated mechanism is shown as (Figure 12).

## 4. Materials and Methods

### 4.1. Preparation of Cell

SH-SY5Y cells were obtained from the American Type Culture Collection (No. CRL-2266) and were cultured in Dulbecco’s modified Eagle’s medium (DMEM) (Invitrogen Corporation, Carlsbad, CA, USA) supplemented with 10% heat-inactivated fetal bovine serum (FBS), 2 mM glutamine, 1 mM pyruvate, 4.5 g/L glucose, 50 U/mL penicillin, and 50 μg/mL streptomycin at 37 °C in a humidified incubator (thermoelectron corporation, Waltham, MA, USA) with 5% CO_2_:95% air under standard conditions. In all experiments, cells were acclimated for 24 h before treatment.

### 4.2. Neuroprotective Analysis

The neuroprotective analysis was conducted according to previous study [80,81]. In brief, cell survival was determined after treatment with Alamar blue, a tetrazolium dye. This assay is similar in principle to the 3-(4,5-dimethyldiazol-2-yl)-2,5-diphenyl tetrazolium bromide cell viability assay and has been previously validated as an accurate measure of the survival of SH-SY5Y cells [82]. Relative protection (percent) was calculated as 100× [(optical density (OD) of 6-OHDA/1T3O-treated cells−OD of 6-OHDA-treated cells)/(OD of control cells−OD of 6-OHDA-treated cells)] [80].

### 4.3. Cell Morphology

SH-SY5Y cells were seeded at a density of 2 × 10^6^ in a 6-cm dish for 24 h before drug treatment [83]. Cells were divided into four groups depending on the drug treatment and include: negative control without drug; 6-OHDA group (6-OHDA only (20 μM)); 1T3O + 6-OHDA group (6-OHDA (20 μM) added 1 h after pretreatment with 1T3O (1 μM)); and 1T3O group (1 h pretreatment with 1T3O (1 μM)). Cells were incubated for 8 h after 6-OHDA treatment and observed using an inverted microscope. (DMI-3000B, Leica, Wetzlar, Germany).

### 4.4. Analysis of Cellular Oxidative Stress (CellROX^®^ Stain)

The CellROX^®^ Oxidative Stress Reagent (Life Technologies, Carlsbad, CA, USA) is a cell-permeable dye that shows a green fluorescence after oxidizing by reactive oxygen species (ROS) and binding to DNA. SH-SY5Y cells were seeded in a 6-cm dish for 24 h before drug treatment [83]. Cells were pretreated with 1T3O for 1 h before 6-OHDA (20 μM) was added. After cells were incubated for 2 h, 5-μM CellROX^®^ Green Reagent was added to the cell culture media and the plate was incubated at 37 °C for 30 min. Next, cells were washed with phosphate buffered saline (PBS) three times and 4′,6-diamidino-2-phenylindole (DAPI), a nuclear stain, was added before 10 min of incubation at 37 °C. An inverted fluorescent microscope (Leica, DMI 3000 B) was used to study the ROS status of cells.

### 4.5. Hoechst Stain

The Hoechst stain is a cell-permeable fluorescent dye that enters apoptotic cells and binds chromatin DNA for fluorescent detection of apoptosis. SH-SY5Y cells were seeded in a 96-well plate (3 × 10^4^ cells/well) and incubated for 24 h before drug treatment [83]. Cells were pretreated with 1T3O for 1 h before 6-OHDA (20 μM) was added. The plate was then incubated for 15 h and apoptosis was assessed using the Hoechst stain (CaspaTag™ Caspase 3, 7 In Situ Assay Kit (Chemicon International, Temecula, CA, USA). Briefly, 0.5 μL Hoechst stain (0.5% *v*/*v*) was added into the culture media and allowed to react for 5 min. After washing the cells twice with a wash buffer, a fixative was added and apoptosis was evaluated using a fluorescent microscope (Leica, DMI-3000B). The apoptosis ratio was calculated on the basis of the method proposed by Zhao et al. in 2007 [84]. Five random fluorescent images and five corresponding optical images of the same positions were taken using an inverted fluorescent microscope. The apoptosis ratio was calculated by dividing the number of apoptotic cells by the number of total counted cells. The mean was calculated and taken as the actual apoptotic ratio [84].

### 4.6. TUNEL Stain

TUNEL stain can be used to study cell apoptosis by fluorescent labeling of the site of chromosomal DNA fragmentation. SH-SY5Y cells were seeded onto a 6-well culture plate (1.8 × 10^6^ cells/well) with coverslips (24 × 24 mm) and cultured for 24 h before drug treatment [83]. Cells were preincubated with the drug for 1 h before 6-OHDA (20 μM) was added. After 8 h of incubation, the In Situ Cell Death Detection Kit, POD (TUNEL assay, Roche Diagnostics GmbH, Mannheim, Germany) was used according to the manufacturer’s instructions. After washing the wells twice with PBS, 4% paraformaldehyde solution in PBS was added for fixation for 1 h. Wells were washed once with PBS and a 3% H_2_O_2_ solution in methanol was added for 10 min for blocking. The wells were washed again with PBS and placed on ice before the addition of the permeabilization solution. After incubation for 2 min, the wells were washed with PBS twice and the TUNEL reaction mixture was added. The plate was sealed with paraffin and incubated in a 37 °C incubator for 1 h. Next, the wells were washed with PBS three times before the addition of DAPI. After 10 min of incubation in a 37 °C incubator, the coverslips were removed and mounted onto glass slides with an adhesive. The images were examined using a Leica, DM 6000 B microscope and captured using a SPOT Xplorer Digital camera (Diagnostic Instruments Inc., Sterling Heights, MI, USA). The apoptosis ratio was calculated on the basis of the method proposed by Zhao et al. in 2007: randomly, five fluorescent images were captured using the inverted fluorescent microscope, together with five images of DAPI-stained nuclei from the same positions [84]. After the cells were counted, the number of apoptotic cells was divided by the total number of cells to obtain the apoptosis ratio. The mean of five images were taken as the actual cell apoptosis ratio [84].

### 4.7. Oxidative Stress (SOD Activity Assay)

Water-soluble tetrazolium salts-1 (WST-1) can produce a water-soluble formazan dye upon reduction with superoxide ions (O_2_^−^). As this reaction can be inhibited by SOD, colorimetric analysis of the product of the WST-1 reaction can be used to determine SOD enzyme activity. SH-SY5Y cells were seeded onto 6-cm culture dish and incubated for 24 h before drug treatment [83]. The cells were pretreated with 1T3O for 1 h before 6-OHDA (20 μM) was added. After 24 h of treatment, a cell scraper was used to remove the cells that were attached to the surface of the dish. The cells and culture media were transferred to a centrifuge tube and centrifuged for 8 min at 3000 rpm, 4 °C. The culture media was aspirated and a 4 °C lysis buffer (0.5% Triton X-100, 5-mM β-ME and 0.1 mg/mL phenylmethylsulfonyl fluoride, PMSF) was added. The mixture was centrifuged at 14,000 rpm for 5 min before the protein supernatant was removed. According to the protein quantification method (Bio-Rad, Hercules, CA, USA) by Lowry et al. [85], bovine serum albumin was employed as the standard and a DC protein assay kit (Bio-Rad, Hercules, CA, USA) was used to construct a standard curve from the absorbance values. Sample protein concentration was determined by interpolation. All samples were adjusted to the same protein concentration using the lysis buffer and the SOD activity assay kit (Biovision, Exton, PA, USA) was used to measure intercellular SOD activity. Absorbance values were measured using an ELISA reader (Bio Tek Instruments, Inc., Winooski, VT, USA) and the SOD inhibition rate was calculated according to the manual included with the SOD activity assay kit:

SOD activity (inhibition rate %) = [(Blank1 − Blank3) − (sample − Blank2)]/(Blank1 − Blank3) × 100

### 4.8. Fish Maintenance

The AB strain of *zebrafish* embryos was collected after natural spawning, staged according to standard criteria, and raised in Hank’s buffer (13.7 mM NaCl, 540 μM KCl, 25 μM Na_2_HPO_4_, 44 μM KH_2_PO_4_, 300 μM CaCl_2_, 100 μM MgSO_4_, 420 μM NaHCO_3_, and pH 7.4) at 28.5 °C. Because the *zebrafish* embryos receive nourishment from the attached yolk sac, no additional maintenance was required.

### 4.9. Locomotor Behavioral Test

The locomotor behavior test was performed as previously described [72]. The zebrafish embryos at 9 hpf (hours post fertilization) were treated with 1T3O for 4 days either in the presence or in the absence of 6-OHDA in a 24-well plate. The zebrafish larvae were transferred into 10-cm dishes (16 fish/dish) at 5 dpf (days post fertilization), and the swimming behavior test was then monitored by an animal behavior system with an automated video tracking (Singa Technology Co. Taipei, Taiwan; catalog No. TM-01) [86]. The zebrafish larvae were then transferred into a quartz cuvette, which was 4.5 cm in height with a width and length of 1 cm, used for this experiments. The cuvette was housed in a distinctive plastic box that was 16 cm long and 4.8 cm wide. The cuvette was placed in front of a camera (Weichu Technology Co., Ltd., Taipei, Taiwan; catalog No. IC-200) at a distance of 7.5 cm. All instruments were adhered to a plastic plate that was 38 cm long and 19 cm wide. Four cuvettes were placed in a parallel arrangement during a test. Individual zebrafish larvae were put into the tank gently and then tracked from the side of the tank so that it was possible to determine the swim height. Each fish was given a 2-min adaptation period, and the swimming pattern was then recorded for 5 min. The total distance was defined as the distance (in cm) that the fish moved during one 5 min session. 

### 4.10. Western Blot Analysis

#### 4.10.1. Protein Extraction from Cultured Cells

SH-SY5Y cells were pretreated with 1T3O for 1 h and then exposed to 20 μM 6-OHDA. For detecting phosphorylation of Akt, ERK, and p38, SH-SY5Y cells were pretreated with 1T3O for 1 h and then treated with 20 μM 6-OHDA at 15, 30, 60, and 120 min separately. For HO-1 activation detection, SH-SY5Y cells were pretreated with 1T3O for 1 h and then treated with 20 μM 6-OHDA for 24 h. For caspase-3 cleavage detection, SH-SY5Y cells were pretreated with 1T3O for 1 h and then treated with 20 μM 6-OHDA for 8 h. The cells were then washed with ice-cold phosphate-buffered saline (PBS), and lysed in ice-cold lysis buffer (50 mM Tris, pH 7.5, 150 mM NaCl, 1% Triton X-100, 100 μg/mL phenylmethylsulfonyl fluoride, 1 μg/mL aprotinin). The resulting homogenate was centrifuged at 20,000× *g* for 30 min at 4 °C. The supernatant was decanted carefully from the pellet, and analyzed as the total protein extract. The supernatant was then reserved for Western blot analysis. Protein concentrations were determined using the DC protein assay kit (Bio-Rad, Hercules, CA, USA) modified from the method of Lowry et al. [86].

#### 4.10.2. Preparation of Nuclear Extracts

For Nrf2 activation detection, SH-SY5Y cells were pretreated with 1T3O for 1 h and then treated with 20 μM 6-OHDA for 3 h. The extraction and isolation of nuclear fractions were performed with the nuclear extraction kit (Millipore, Billerica, MA, USA), according to the manufacturer’s instructions. The procedure is based on cell lysis with mild detergents. The prepared cell extracts were compatible with Western blotting.

#### 4.10.3. Protein Extraction from Zebrafish

Zebrafish embryos at 9 hpf (hours post fertilization) were treated with 1T3O until reaching 4 dpf (days post fertilization) either in the presence or in the absence of 6-OHDA. Zebrafish larvae were then washed with ice-cold PBS, homogenized on ice using a sonicator in ice-cold lysis buffer (50 mM Tris (pH 7.5), 150 mM NaCl, 1% Triton X-100, 100 μg/mL phenylmethylsulfonyl fluoride, and 1 μg/mL aprotinin), and then centrifuged at 20,000× *g* for 30 min at 4 °C. The supernatant was collected carefully and analyzed as the total protein extract. The supernatant then was used for Western blotting. Protein concentrations were measured using the DC protein assay kit (Bio-Rad), which was modified from the method of Lowry et al. [86].

#### 4.10.4. Western Blotting

Western blotting was performed as previously described [87]. The supernatants containing protein extraction from SH-SY5Y cells and zebrafish larvae were mixed with equal volumes of sample buffer (2% sodium dodecyl sulfate (SDS), 10% glycerol, 0.1% bromophenol blue, 2% 2-mercaptoethanol, and 50 mM Tris-HCl, pH 7.2). The mixture was then loaded onto a tricine SDS-polyacrylamide gel and electrophoresed at 150 V for 90 min. The proteins were transferred to a polyvinylidene difluoride membrane (PVDF; Immobilon-P, Millipore, 0.45-μM pore size) at 125 mA overnight at 4 °C in transfer buffer (50 mM Tris-HCl, 380 mM glycine, 1% SDS, and 20% methanol). After blocking for 40 min with 5% non-fat dry milk in Tris-buffered saline (TTBS; 0.1% Tween 20, 20 mM Tris-HCl, 137 mM NaCl, pH 7.4), the membrane was incubated for 120 min at room temperature with the following primary antibodies: p-Akt (Ser473, catalog 9271), Akt (catalog 9272), p-ERK (Thr202/204, catalog 9101), ERK (catalog 9102), p-p38 (Thr180/Thr182, catalog 9211), p38 (catalog 9212), HO-1 (catalog 3162) (all from Cell Signalling Technology, Danvers, MA, USA); caspase-3 (catalog IMG-144A, Imgenex, San Diego, CA, USA); lamin B1 (catalog Ab616048), Nrf2 (Ab31163) (both from Abcam, Inc., Cambridge, UK); TH (catalog MAB318, EMD Millipore Corporation, Princeton, NJ, USA); and TNF-α (catalog ARC3012, Invitrogen Corporation, Waltham, MA, USA). After the membrane was washed three times for 10 min each with TTBS buffer, it was incubated in the appropriate horseradish peroxidase-conjugated (HRP-conjugated) secondary antibody at room temperature for 120 min. Images were obtained using the UVP BioChemi Imaging System (Upland, CA, USA). Furthermore, the relative densitometry was quantified by using LabWorks 4.0 software (UVP Bioimaging Systems, Upland, CA, USA). The membranes were reprobed with monoclonal antibodies against β-actin (catalog GTX124500, GeneTex, Inc., Irvine, CA, USA) as the internal control for protein loading.

### 4.11. Data Analysis

All experimental data were presented by mean ± SEM. One-way analysis of variance was used for statistical analysis and Duncan’s method was used to test the significance of the differences among the different means.

## 5. Conclusions

The present study demonstrates that 6-OHDA-stimulated SH-SY5Y cells and zebrafish were in vitro and in vivo model, respectively, for evaluating the neuroprotective effects of compounds. The marine-related compound, 1-tosylpentan-3-one (1T3O), was able to regulate the expression of Akt, ERK, p38 MAPK, and Nrf2/HO-1 protein expression in 6-OHDA-challenged SH-SY5Y cells. In addition, pretreatment of 1T3O significantly inhibited 6-OHDA-induced deficits in locomotor activity in zebrafish larvae. Moreover, our Western blot results showed that 1T3O significantly inhibited 6-OHDA-induced upregulation of TNF-α protein and downregulation of TH protein expressions in the heads of zebrafish larvae. In summary, 1T3O attenuated 6-OHDA-induced neurotoxicity in vitro and in vivo.

## Figures and Tables

**Table 1:** Effect of 1-tosylpentan-3-one (1T3O) on zebrafish mortality. Zebrafish embryos were treated with 1T3O at concentrations of 10^2^, 50, 5, 5 × 10^−1^, 5 × 10^−2^, and 5 × 10^−3^ μM 9 h to 4 days after fertilization, and zebrafish mortality was recorded. Results show that at concentrations of 50, 5, 5 × 10^−1^, 5 × 10^−2^, and 5 × 10^−3^ μM, no mortality was observed.

Concentration (μM)	Mortality Count	Mortality (%)
Control	0/24	0
100	24/24	100
50	0/24	0
5	0/24	0
5 × 10^−1^	0/24	0
5 × 10^−2^	0/24	0
5 × 10^−3^	0/24	0

## Abbreviations

PD	Parkinson’s disease
ND	neurodegenerative disease
6-OHDA	6-hydroxydopamine
1T3O	1-tosylpentan-3-one
TNF-α	tumor necrosis factor-alpha
cAMP	cyclic adenosine monophosphate
CRE	cAMP responsive element
CREB	cAMP responsive element binding protein
PI3K	phosphoinositide 3-kinase
Akt	protein kinase B
ERK	extracellular signal–regulated kinase
MAPK	mitogen-activated protein kinase
Nrf2	nuclear factor erythroid 2–related factor 2
HO-1	heme oxygenase-1
TUNEL	terminal deoxynucleotidyl transferase dUTP nick end labeling
Bcl-xL	B-cell lymphoma-extra-large
d-UTP	2´-deoxyuridine 5´-triphosphate
DAPI	4′,6-diamidino-2-phenylindole
SOD	superoxidase dismutase
ZnPP	zinc protoporphyrin
ARE	antioxidant response element
NQO1	NAD(P)H:quinone-oxidoreductase-1
NAD(P)H	lactaldehyde reductase
GSHP	glutathione peroxidase
PGE2	prostaglandin E2
WST-1	Water-soluble tetrazolium salts-1
HRP	horseradish peroxidase
MAO-B	monoamine oxidase B
EGCG	epigallocatechin gallate
MPP^+^	1-methyl-4-phenylpyridinium
MPTP	1-methyl-4-phenyl-1,2,3,6-tetrahydropyridine
